# Elucidating the Skin Delivery of Aglycone and Glycoside Flavonoids: How the Structures Affect Cutaneous Absorption

**DOI:** 10.3390/nu9121304

**Published:** 2017-11-30

**Authors:** Shih-Yi Chuang, Yin-Ku Lin, Chwan-Fwu Lin, Pei-Wen Wang, En-Li Chen, Jia-You Fang

**Affiliations:** 1 Research Center for Food and Cosmetic Safety and Research Center for Chinese Herbal Medicine, Chang Gung University of Science and Technology, Kweishan, Taoyuan 333, Taiwan; clemencechuang@gmail.com (S.-Y.C.); cflin@mail.cgust.edu.tw (C.-F.L.); 2 Department of Traditional Chinese Medicine, Chang Gung Memorial Hospital, Keelung 204, Taiwan; lin1266@cgmh.org.tw; 3 School of Traditional Chinese Medicine, Chang Gung University, Kweishan, Taoyuan 333, Taiwan; 4 Department of Cosmetic Science, Chang Gung University of Science and Technology, Kweishan, Taoyuan 333, Taiwan; 5 Department of Medical Research, China Medical University Hospital, China Medical University, Taichung 404, Taiwan; pwwang@hotmail.com; 6 Pharmaceutics Laboratory, Graduate Institute of Natural Products, Chang Gung University, Kweishan, Taoyuan 333, Taiwan; fajy5521@gmail.com; 7 Chinese Herbal Medicine Research Team, Healthy Aging Research Center, Chang Gung University, Kweishan, Taoyuan 333, Taiwan; 8 Department of Anesthesiology, Chang Gung Memorial Hospital, Kweishan, Taoyuan 333, Taiwan

**Keywords:** flavonoid, cutaneous absorption, structure–permeation relationship, anti-inflammation, molecular modeling

## Abstract

Flavonoids are bioactive phytochemicals that exhibit protective potential against cutaneous inflammation and photoaging. We selected eight flavonoid aglycones or glycosides to elucidate the chemistry behind their skin absorption capability through experimental and computational approaches. The skin delivery was conducted using nude mouse and pig skins mounted on an in vitro Franz cell assembly. The anti-inflammatory activity was examined using the O_2_^•–^ and elastase inhibition in activated human neutrophils. In the equivalent dose (6 mM) application on nude mouse skin, the skin deposition of naringenin and kaempferol was 0.37 and 0.11 nM/mg, respectively, which was higher than that of the other flavonoids. Both penetrants were beneficial for targeted cutaneous therapy due to their minimal diffusion across the skin. The absorption was generally greater for topically applied aglycones than glycosides. Although naringenin could be classified as a hydrophilic flavonoid, the flexibility of the chiral center in the C ring of this flavanone could lead to better skin transport than the flavonols and flavones with a planar structure. An optimized hydrophilic and lipophilic balance of the flavonoid structure was important for governing the cutaneous delivery. The hydrogen bond acceptor and stratum corneum lipid docking estimated by molecular modeling showed some relationships with the skin deposition. The interaction with cholesteryl sulfate could be a factor for predicting the cutaneous absorption of aglycone flavonoids (correlation coefficient = 0.97). Baicalin (3 µM) showed the highest activity against oxidative burst with an O_2_^•–^ inhibition percentage of 77%. Although naringenin displayed an inhibition efficiency of only 20%, this compound still demonstrated an impressive therapeutic index because of the high absorption. Our data are advantageous to providing the information on the structure–permeation relationship for topically applied flavonoids.

## 1. Introduction

Strong evidence links oxidative stress with several pathologies [1]. The skin, the largest organ in the body, is a main target of oxidative stress because of its size [2]. Reactive oxygen species (ROS) play a key role in some cutaneous diseases, including photoaging, psoriasis, dermatitis, acne, rosacea, alopecia, and skin cancer [3]. Flavonoids are antioxidants ubiquitously found in vegetables and fruits. The structure of flavonoids is associated with the derivatives of chalcone. They can prevent or treat cutaneous inflammation and malignancy by maintaining the skin’s homeostasis [4]. Some flavonoids, such as naringenin and quercetin, have been reported to exhibit anti-aging activity that slows down the skin’s natural senescence [5]. Flavonoids have been largely used in dermatology and cosmeceuticals in the form of crude extracts.

**Figure 1:**
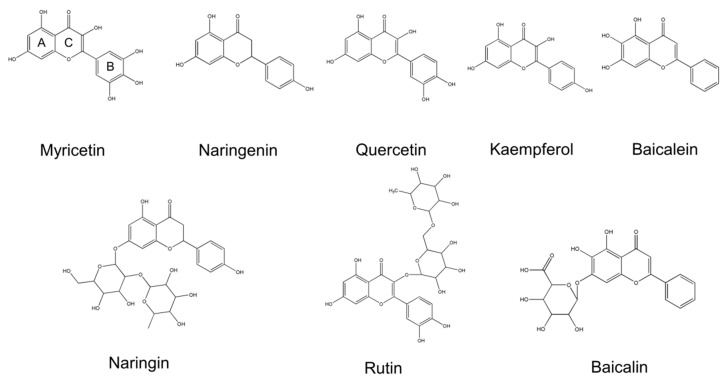
The chemical structures of flavonoids tested in this study.

Topical application provides an efficient method for facilitating the local action of drugs on the skin [6]. For an active ingredient to exhibit bioactivity, its delivery into the targeted skin must be successful. It is evidenced that the absorption rate and scavenging activity of some flavonoids depend on their chemical structure [7]. The exploration of the structure–permeation relationship (SPR) offers insights into the understanding of how the physicochemical properties of chemicals influence cutaneous absorption or targeting. The establishment of SPR is useful for predicting the skin delivery of the compounds and for facilitating the development of new topically applied actives. In addition to the aglycone form, flavonoids are present in the form of glycoside in nature. Some aglycones and glycosides, such as quercetin/rutin and baicalein/baicalin, are paired. Glycosides also show potent bioactivity in many cases. For example, baicalin is proven to protect the skin from UV radiation, burning, and aging [8]. Although many reports have dealt with the biological effect of glycoside flavonoids, investigation of how the sugar moiety affects cutaneous absorption is still lacking. In a continuing attempt to elucidate SPR, the objective of this study was to evaluate the physicochemical characteristics and the skin absorption of a series of aglycone flavonoids and their corresponding glycosides. These included myricetin, naringenin, quercetin, baicalein, kaempferol, naringin, rutin, and baicalin (Figure 1).

In the present study, the in vitro Franz cell model was utilized to assess the skin absorption of flavonoids. Both nude mouse skin and baby pig skin were employed as the permeation barriers. In inspecting the permeation behavior of a penetrant, it is important to identify the possible transport pathways [9]. We examined flavonoid permeation via the skin after removal of the stratum corneum (SC), lipid, sebum, and protein in order to understand the delivery routes. Data collected for the capacity factor (log *K*’), the partition coefficient (log *P*), and the aqueous solubility allowed us to distinguish the relative contribution to cutaneous absorption. The anti-inflammatory activity of these compounds was determined by the capacity of inhibiting superoxide anion (O_2_^•–^) and elastase of formyl-methionyl-leucyl phenylalanine (fMLF)-activated human neutrophils. A wealth of information on the hydrogen bond acceptor, the hydrogen bond donor, the total polarity surface, and the molecular volume was obtained by the computation of molecular modeling. We also investigated the predicted interaction between flavonoids and SC lipid components to explain the skin permeation trend. The present study sheds light on the effect of the flavonoid structure on skin absorption and targeting, broadening the comprehension of SPR in natural antioxidants.

## 2. Materials and Methods

### 2.1. Materials

All flavonoids tested in this study were purchased from Sigma-Aldrich (St. Louis, MO, USA). All other chemicals and solvents were of reagent grade without further purification.

### 2.2. Capacity Factor (Log K’)

The HPLC setup for the calculation of log *K*’ was an HPLC system (7-series, Hitachi, Tokyo, Japan) with a LiChrospher^®^ C18 column (200 × 4.6 mm, Merck, Darmstadt, Germany). The mobile phase consisted of a mixture of methanol and pH 2 phosphate buffer solution (1:1). The flow rate and wavelength for determination were 1 mL/min (L-7110 pump) and 256 nm (L-7455 diode array detector), respectively. The retention times of flavonoids were detected, and the log *K*’ was computed as log [(t_r_ − t_0_)/t_0_], where t_r_ and t_0_ were the retention time of the flavonoid and the nonretained solvent peak, respectively.

### 2.3. Partition Coefficient (Log P)

Flavonoids in methanol (0.5 mg/mL) were pipetted into a tube at a volume of 1 mL. Methanol was then evaporated under vacuum. In the amount of 1 mL of each, *n*-Octanol and water were incorporated into the tube. After being shaken for 24 h at 37 °C, the tube was centrifugated at 10,000× *g* for 10 min. The flavonoid content in both phases was analyzed by HPLC. The log *P* was calculated as log (flavonoid content in *n*-octanol/flavonoid content in water).

### 2.4. Saturated Solubility in 20% PEG400 Aqueous Solution

The saturated solubility of flavonoids in 20% PEG400/pH 7.4 buffer was measured by loading 20 mM compounds in the vehicle and shaking them at 37 °C for 2 h. The suspension was then centrifuged at 10,000× *g* for 10 min. The supernatant was filtered across the polyvinylidene fluoride membrane with a pore size of 0.45 µm. The resulting supernatant was analyzed by HPLC to record the solubility (mM).

### 2.5. Animals

Eight-week-old female nude mice were provided by the National Laboratory Animal Center (Taipei, Taiwan). One-week-old specific-pathogen-free pigs were obtained from the Animal Technology Institute Taiwan (Miaoli, Taiwan). All animals were treated in strict accordance with the recommendations set forth in the Guidelines for the Institutional Animal Care and Use Committee of Chang Gung University (CGU15-083).

### 2.6. Preparation of SC-Disrupted Skin

The full-thickness dorsal skin of the nude mouse was excised after sacrifice. The SC-stripped skin was obtained by stripping the skin surface 20 times with adhesive tape (Scotch^®^, 3M, Maplewood, MN, USA). The delipidized skin was achieved by incubating the skin surface onto a chloroform/methanol solution (2:1) for 2 h [10]. To prepare the desebumed skin, the SC side of the skin was washed with cold hexane (4 °C) five times, based on the procedure of the previous study [11]. The skin’s surface was then treated with a 40% ethanol/water solution for 2 h, a process that successfully denatured the skin’s proteins.

### 2.7. In Vitro Franz Cell Assembly

The cutaneous absorption of flavonoids was assessed using a Franz diffusion cell system. The excised animal skin or cellulose membrane was mounted between the donor and receptor compartments with the SC facing up toward the donor. The receptor was filled with 30% ethanol/pH 7.4 buffer and placed on a magnetic stirring plate. The effective permeation area of the cell was 0.785 cm^2^. The stirring rate and temperature of the receptor were 600 rpm and 37 °C, respectively. Saturated solution or a 6 mM flavonoid suspension was prepared in 20% PEG400/pH 7.4 buffer to be loaded into the donor compartment. A 300-µL aliquot in the receptor was collected at determined durations, followed by an immediate replacement with fresh receptor medium. The amount of flavonoid in the receptor was determined by HPLC. The skin was removed from the cell after a 24-h application. After being washed with water, the skin sample was weighed and positioned in a vial with 1 mL methanol. MagNA Lyser (Roche) was used to homogenize the skin. The homogenate was centrifuged at 10,000× *g* for 10 min. The supernatant was analyzed by HPLC to quantify the flavonoid deposition in the skin reservoir.

### 2.8. Inhibition of Neutrophilic Inflammation by Flavonoids

The protocol was approved by the Institutional Review Board at Chang Gung Memorial Hospital, and written informed consent was obtained from all volunteers (201600500B0C101). Whole blood was withdrawn from healthy volunteers between 20 and 30 years of age. Human neutrophils were isolated using a typical method of dextran sedimentation prior to centrifugation in a Ficoll-Hypaque gradient and hypotonic lysis of erythrocytes [12]. The granulocyte layer was harvested and suspended in calcium-free HBSS at pH 7.4, which was maintained at 4 °C until use.

The reduction of ferricytochrome *c* was utilized for measuring the superoxide anion (O_2_^•–^) release from the neutrophils [13]. The human neutrophils (6 × 10^5^ cells/mL) were incubated with 0.5 mg/mL ferricytochrome *c* and 1 mM CaCl_2_ at 37 °C, and were then treated with flavonoids (3 µM) in DMSO for 10 min. Neutrophils were stimulated by adding 0.1 µM fMLF with cytochalasin B (1 µg/mL). The reduction of ferricytochrome *c* was monitored by the absorbance at 550 nm using a U3010 ultraviolet/visible spectrophotometer (Hitachi, Tokyo, Japan). The human neutrophils were equilibrated with an elastase substrate (MeO-Suc-Ala-Ala-Pro-Val-*p*-nitroanilide, 100 µM) at 37 °C for 2 min. Flavonoids at a concentration of 3 µM were added into the neutrophil suspension for 10 min. The cells were activated by fMLF and cytochalasin B for a further 10 min. Elastase release was detected by measuring the change of absorbance at 405 nm.

### 2.9. Molecular Modeling

The structures of the flavonoids were sketched using Discovery Studio^®^ version 4.1 workstation (Accelrys, San Diego, CA, USA). The hydrogen bond acceptor or donor number, total polarity surface, and molecular volume of the flavonoids were estimated. The superimposition of flavonoids with ceramides, palmitic acid, cholesteryl sulfate, and cholesterol was computed to observe the conformation and ligand-binding activity. The negative CDOCKER energy was calculated after conducting the molecular docking simulation of the flavonoids with these SC lipids.

### 2.10. In Vivo Cutaneous Tolerance of Flavonoids

The 20% PEG400/pH 7.4 buffer loaded with flavonoids at 6 mM was topically applied daily (0.6 mL) on the nude mouse back for 7 days. The flavonoid-containing vehicle was replaced with a new one each day. After removal of the vehicle, the treated skin region was evaluated by Tewameter TM300 (Courage and Khazaka, Köln, Germany) to determine the transepidermal water loss (TEWL). Stained by hematoxylin and eosin (H&E), the treated skin was excised for histological examination after a 7-day flavonoid application.

### 2.11. Data Analysis

In the cutaneous absorption experiment, flavonoid deposition in the skin was estimated as the molar amount per mg of skin (nM/mg). In the case of the skin deposition from the saturated solution, the calibrated skin deposition (CSD) was measured as flavonoid deposition divided by the applied dose (saturated solubility). The flux (nM/cm^2^/h) was estimated by the slope of the penetrated amount–time curve. The permeability coefficient (PC) was calculated from the flux divided by the saturated solubility of the flavonoids. The dermal/transdermal selectivity index (*S* value) was calculated using an equation of skin deposition/flux (for equivalent dose application) or CSD/PC (for saturated dose application).

The data in the present study were presented as mean and standard deviation (S.D.). The statistical difference in the data of different experimental groups was evaluated using the Kruskal–Wallis test. The post hoc test for checking individual differences was Dunn’s test. A 0.05 level of probability was taken as the statistical significance. The software used for statistical comparison was WINKS (Texasoft, Cedar Hill, TX, USA).

## 3. Results

### 3.1. Physicochemical Properties of Flavonoids

To understand the relationship between skin permeation and physicochemical properties, the molecular weight (MW), lipophilicity (log *K*’ and log *P*), and aqueous solubility of flavonoids were considered as presented in Table 1. Figure 1 demonstrates that the aglycone flavonoids tested in this study have different hydroxyl groups from 3 to 6, with myricetin showing the most abundant hydroxyl moieties and the highest MW. The structures of most aglycones belong to flavonols, with the exception of the flavanone naringenin and flavone baicalein. Naringin, rutin, and baicalin are the glycosides with sugar residue in the structures of naringenin, quercetin, and baicalein, respectively. The glycosides had a higher MW than their corresponding aglycones. Both log *K*’ and log *P* are the indicators of lipophilicity. The capacity factor was increased following the decrease of hydroxyl moieties for flavonols and flavones. The trend of log *P* correlated well with that of log *K*’. Although kaempferol exhibited a higher partition coefficient compared to baicalein (3.71 versus 3.59), no statistically significant difference was found (*p* > 0.05). Naringenin, a flavanone, revealed a lower lipophilicity than baicalein, though both compounds possessed the same hydroxyl group number (3). The lipophilicity of glycosides was less than that of aglycones because of the presence of sugar. As expected, the compounds with lower lipophilicity displayed higher solubility in PEG400/pH 7.4 buffer solution. The flavanone showed much higher solubility compared to the flavonols and flavones. Naringenin was 470-fold more soluble than baicalein. The same result was achieved with naringin, which showed an aqueous solubility of >20 mM.

### 3.2. Cutaneous Absorption of Flavonoids

**Figure 2:**
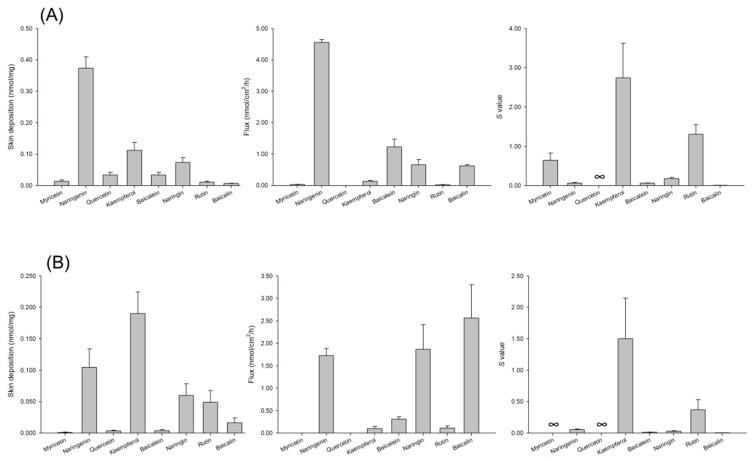
Skin deposition, flux, and *S* value of flavonoids at a dose of 6 mM after topical treatment on nude mouse and pig skins: (**A**) nude mouse skin; and (**B**) pig skin. The donor vehicle is 20% PEG400 in pH 7.4 buffer. All data are presented as the mean of four experiments ± S.D.

The cutaneous absorption of the penetrants was compared using a Franz cell assembly. Nude mouse and baby pig dorsal skins were used in this experiment due to their close similarity to human skin. The donor dose was first set at an equivalent concentration (6 mM). This dose surpassed the saturated solubility. This led to a condition of suspension in the donor except for naringin, which showed a solution type because of the extremely high solubility in the aqueous vehicle. Both flavonoid deposition within the skin and the flux across the skin were examined in this study. Dermal delivery should be addressed in the skin deposition that is carried out with the aim of cutaneous targeting with minimizing systemic absorption. Flux is a factor to use in predicting how well the penetrant will reach the systemic circulation. Figure 2A,B depicts the skin permeation profiles of flavonoids via nude mouse skin and pig skin, respectively. Both animal skins demonstrated similar trends of cutaneous absorption. Among the aglycones tested, naringenin and kaempferol showed the greatest skin deposition. Our results showed that myricetin, which had the lowest lipophilicity among the flavonoids examined, was the aglycone with the least deposition. The glycosides may be susceptible to enzymatic hydrolysis in skin tissue with aglycone release. After HPLC analysis, we found no aglycone in the skin deposition and receptor after topical application of glycosides. This indicated that the glycosides were not metabolized in this case. The skin deposition of the glycosides was minor compared to that of the corresponding aglycones.

Naringenin revealed a high cutaneous delivery not only in skin deposition but also in flux. The highest flux of aglycones was observed with naringenin, followed by baicalein. Myricetin and quercetin levels in the receptor were negligible or even below the HPLC detection limit, giving a flux of near zero. Contrary to the result with skin deposition, glycosylation generally increased the flux, except in the case of naringin diffusion across nude mouse skin. The flux of naringin and baicalin was comparable and significantly higher than that of rutin. Cutaneous targeting is a strategy for local skin prevention or therapy with reduced systemic effects. As shown in Figure 2, we calculated *S* value as an index of selectivity between cutaneous targeting and transdermal delivery. Kaempferol demonstrated the highest *S* value (2.7 and 1.5 for nude mouse skin and pig skin); Myricetin and quercetin were absorbed into the skin reservoir without reaching the receptor, leading to the infinity of *S* value (∞). Rutin was the compound with the highest *S* value (1.3 and 0.4 for nude mouse skin and pig skin, respectively) among the glycosides.

**Figure 3:**
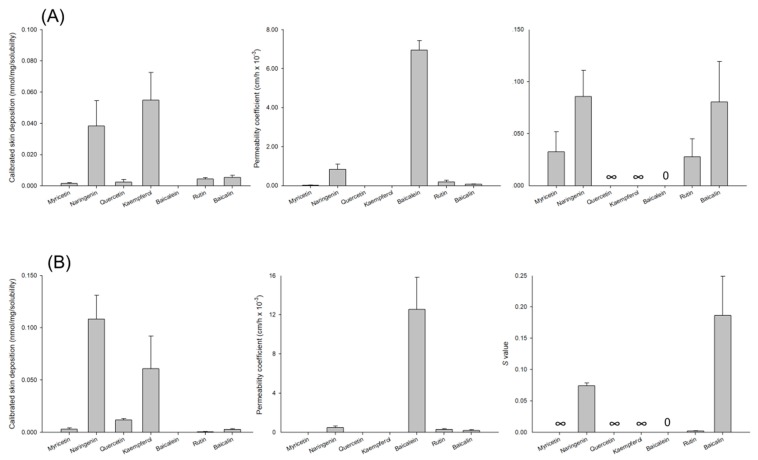
Calibrated skin deposition, permeability coefficient, and *S* value of flavonoids at a dose of saturated solubility after topical treatment on nude mouse and pig skins: (**A**) nude mouse skin; and (**B**) pig skin. The donor vehicle is 20% PEG400 in pH 7.4 buffer. All data are presented as the mean of four experiments ± S.D.

The flavonoids in the donor were also dosed with saturated solubility to achieve the maximum thermodynamic equivalents for all penetrants. Naringin was excluded in this experiment because we could not determine the saturated solubility of this compound (>20 mM). The extremely high solubility would lead to skin damage, complicating the discussion of SPR. The trend of the cutaneous absorption profiles was similar for nude mouse skin and pig skin after topical application of saturated solution, as shown in Figure 3A,B, respectively. Equivalent doses of naringenin and kaempferol showed the highest skin deposition in saturated solubility. The CSD of baicalein was below the quantification limit, which may have been due to the negligible solubility (0.0077 mM). Among the topically applied aglycones, only naringenin and baicalein could penetrate across the skin to the receptor. The neglected PC of myricetin, quercetin, and kaempferol led to the infinity of *S* value. The *S* value of baicalein was 0. The *S* value of baicalin was greater than that of rutin. This result was opposite to the *S* value of glycosides vehiculated at the equivalent dose.

### 3.3. Flavonoid Permeation via SC-Disrupted Skin

**Figure 4:**
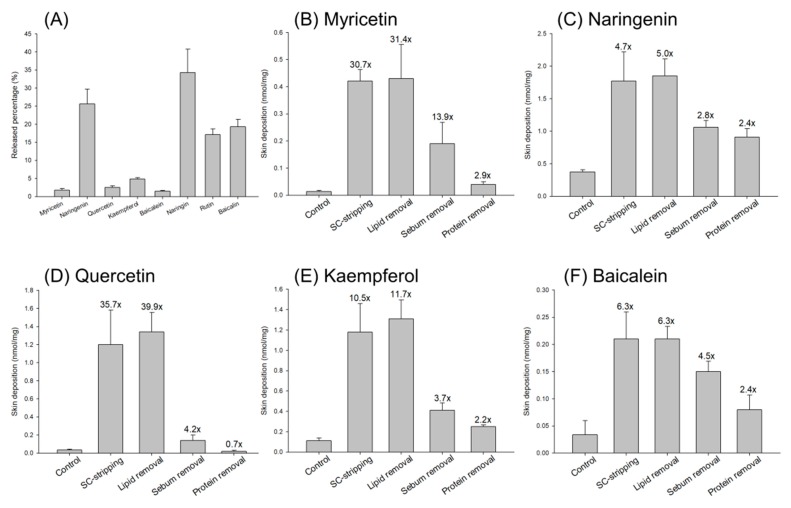

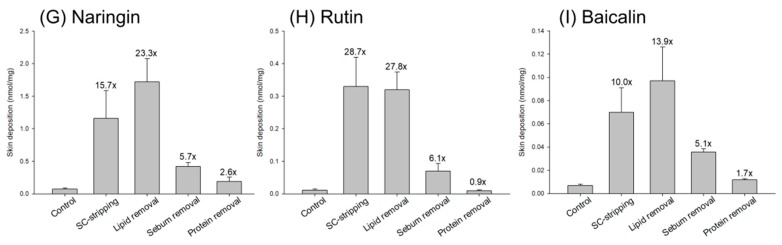
The released percentage and skin deposition of flavonoids (6 mM) via cellulose membrane, stratum corneum (SC)-stripped skin, delipid skin, desebum skin, and deprotein skin: (**A**) released percentage of flavonoids across cellulose membrane; (**B**) skin deposition of myricetin in SC-disrupted skins; (**C**) skin deposition of naringenin in SC-disrupted skins; (**D**) skin deposition of quercetin in SC-disrupted skins; (**E**) skin deposition of kaempferol in SC-disrupted skins; (**F**) skin deposition of baicalein in SC-disrupted skins; (**G**) skin deposition of naringin in SC-disrupted skins; (**H**) skin deposition of rutin in SC-disrupted skins; and (**I**) skin deposition of baicalin in SC-disrupted skins. All data are presented as the mean of four experiments ± S.D.

The SC is the outermost layer of the skin, consisting of the main barrier of most penetrants. Effective permeation through the SC is necessary to obtain successful skin targeting. The SC-disrupted skin was prepared to explore the possible permeation pathways of flavonoids. These included SC-stripped, delipidized, desebumed, and deproteinized nude mouse skins. The penetrants at the equimolar dose (6 mM) were used as the donor in this experiment. Figure 4A demonstrates the released percentage of flavonoids that penetrated the cellulose membrane at 24 h. The release rate was related to the penetrant escape from the vehicle. The release was a delivery stage before penetrant entrance into the SC. The glycosides showed a higher release percentage compared to the aglycones. Naringenin and kaempferol were the two penetrants with greater release than the other aglycones. Figure 4B–I summarizes the cutaneous deposition of eight flavonoids in different SC-disrupted skins. The deposition in SC-stripped skin and delipidized skin was comparable for all flavonoids tested, indicating that intercellular lipid bilayers are an important route for flavonoid transport. The removal of the SC could evoke skin deposition of myricetin and quercetin by >30 fold compared to the intact skin. Myricetin deposition in desebumed skin was 140-fold higher than that in untreated skin. The other flavonoids showed a 2–6-fold increase in skin deposition after sebum removal. The enhancement of skin deposition by protein denaturation was lower than that by lipid removal. The deproteinization process even failed to change the skin deposition of quercetin and its corresponding glycoside. The result of protein removal suggests the minor role of the intracellular route compared to the intercellular route for flavonoid diffusion across the SC.

### 3.4. Inhibition of Neutrophil Inflammation by Flavonoids

The O_2_^•–^ production and elastase release in stimulated neutrophils are indicators reflecting a massive neutrophil infiltration into the inflamed skin. The flavonoids were rated for the inhibition percentage of O_2_^•–^ and elastase in response to fMLF, as shown in Table 2. Incubation of fMLF-activated neutrophils in the presence of flavonoids resulted in an inhibition on superoxide anion, with baicalein and baicalin exhibiting the greatest activity (77% and 76%). The O_2_^•–^ inhibition effect was comparable for the aglycones and the corresponding glycosides. The flavonoids generated less inhibition on elastase than superoxide. Quercetin was among the most potent flavonoids to repress elastase (29%). The elastase inhibition ranged between 10% and 20% for most of the flavonoids. The glycosides had a lower effect on elastase inhibition than the corresponding aglycones. Baicalin did not change the elastase release of fMLF-stimulated neutrophils. To estimate the possible bioactivity after topical delivery, we calculated TI based on multiplying the nude mouse skin deposition and the inflammatory inhibition percentage as shown in Table 2. Although the O_2_^•–^ inhibition of naringenin was weak, this flavanone showed the highest TI because of the preferred delivery into the skin reservoir. The potent activity of baicalein on O_2_^•–^ inhibition had led to a TI of 2.60, which approximated the TI level of kaempferol. The compound with the highest TI for elastase inhibition was also found to be naringenin, followed by kaempferol. Naringin was the penetrant with a higher TI than the other glycosides. Naringin demonstrated less TI value compared to naringenin.

### 3.5. Molecular Modeling

The informative explanation of the effect of molecular structure on cutaneous delivery is based on the hydrogen bond number, the total polarity surface, and the molecular volume. The parameters for flavonoids are computed by Discovery Studio^®^ 4.1 as presented in Table 3. The number of hydrogen bond acceptors and donors increased following the increase of hydroxyl groups in the structure. The hydrogen bond number of glycosides was much higher than that of aglycones due to the presence of hydroxyl moieties in the sugar. Rutin exhibited the highest hydrogen bond numbers among all flavonoids examined. The total polarity surface correlated well with the hydrogen bond number, with the glycosides showing a greater polarity surface than aglycones. This indicates more polar interactions of the glycosides in aqueous medium. The molecular volume is a reflection of MW. We found a high positive correlation between molecular volume and MW (correlation coefficient = 0.9979).

To study the cutaneous transport characteristics of the flavonoids in greater detail, we applied computational molecular docking to analyze the possible interaction of the penetrants to SC lipids. The three-dimensional lipid model was generated for their posterior use as the target structure in the in silico calculation. Table 4 summarizes the best docking score (negative CDOCKER) of the flavonoids interacting with SC lipids, including ceramides II, III, and VI, palmitic acid, cholesteryl sulfate, and cholesterol. The flavonoids were docked into conformationally minimized SC lipids. The cooperativity of the interaction included van der Waals, hydrogen bonding, and lipophilic and electrostatic forces. The greater negative energy dictated a stronger binding interaction. The glycosides revealed greater interaction to ceramides than aglycones, with rutin showing the highest interaction. The discrepancy of the negative CDOCKER between ceramides and different aglycones was not large (−24 to −18).

**Figure 5:**
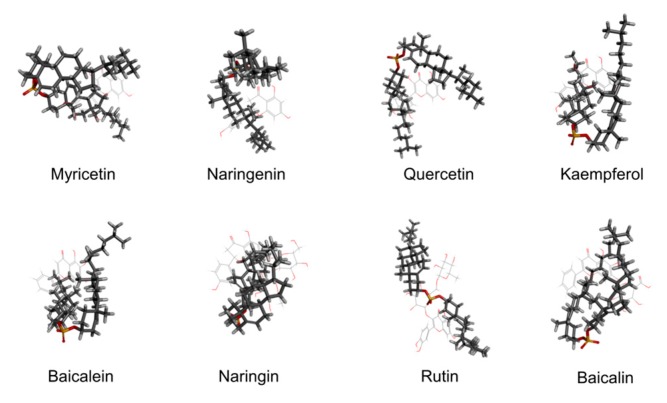
Superimposition of the computed poses for flavonoids with cholesteryl sulfate.

Rutin showed the highest negative CDOCKER level with palmitic acid among the flavonoids detected. A relatively higher negative CDOCKER energy was found for cholesteryl sulfate than for the other lipids. It appears that the flavonoids preferentially interacted with cholesteryl sulfate. In the case of cholesteryl sulfate, the negative CDOCKER of aglycones was higher compared to the corresponding glycosides. Naringenin (−121) and kaempferol (−119) exhibited a more potent interaction with cholesteryl sulfate than the other penetrants. No interaction was detected between cholesterol and the compounds except with rutin and baicalin. Figure 5 illustrates the best docking poses of the flavonoids interacting with cholesteryl sulfate. The flavonoids displayed the ligand binding activity to cholesteryl sulfate but in different conformations.

### 3.6. In Vivo Cutaneous Tolerance of Flavonoids

**Figure 6:**
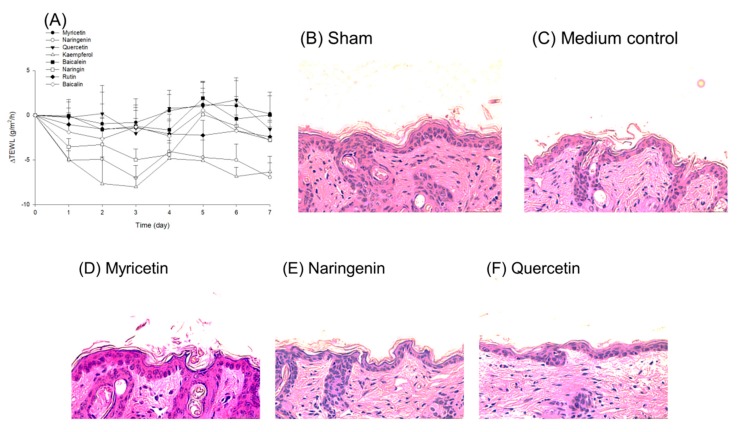

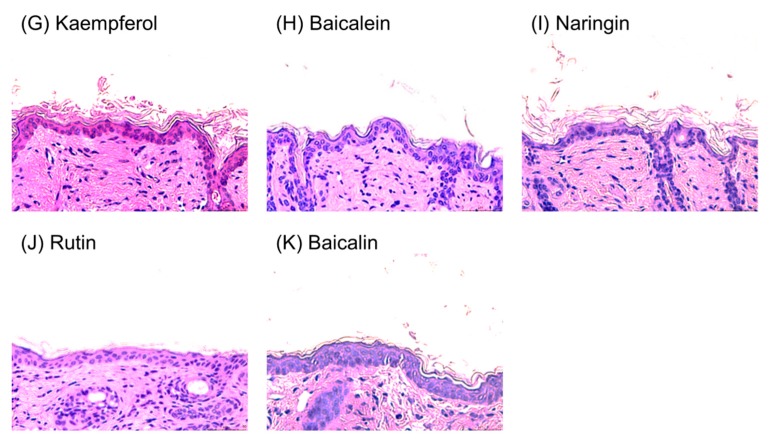
The in vivo safety of topically applied flavonoids on skin after a 7-day exposure: (**A**) ΔTEWL-time curves; (**B**) the histology of sham control skin; (**C**) the histology of aqueous medium-treated skin; (**D**) the histology of myricetin-treated skin; (**E**) the histology of naringenin-treated skin; (**F**) the histology of quercetin-treated skin; (**G**) the histology of kaempferol-treated skin; (**H**) the histology of baicalein-treated skin; (**I**) the histology of naringin-treated skin; (**J**) the histology of rutin-treated skin; and (**K**) the histology of baicalin-treated skin. The TEWL data are presented as the mean of six experiments ± S.D.

The tolerance at the equivalent dose to the skin was determined by in vivo topical exposure for seven days. We examined ΔTEWL, as shown in Figure 6A. ΔTEWL was calculated by the TEWL value of the treated skin area minus the value of the aqueous vehicle control. Most of the flavonoids exhibited a near-zero point ΔTEWL profile. This suggests that these compounds did not induce a barrier defect of SC. Naringenin and kaempferol even reduced TEWL as compared to the vehicle control. This may imply a protective capability of both compounds on barrier function. Figure 6B–K reveals the histology of flavonoid-treated skin after a seven-day administration. We could see a decrease in the SC layers of the vehicle control compared to the sham group (Figure 6B versus Figure 6C), indicating an interruption of barrier position. The SC layers could be recovered through the application of naringenin, kaempferol, and naringin. The flavonoid treatment did not alter the viable skin morphology, expressing a negligible irritation.

## 4. Discussion

Sustained inflammation contributes to the pathogenesis of some skin disorders, including photoaging, psoriasis, and atopic dermatitis. Flavonoids exert potent anti-inflammatory effects on the skin. Nevertheless, whether the cutaneous absorption of flavonoids is sufficient to trigger the bioactivity is questionable. An ideal topical administration has a much higher absorption accomplished in the cutaneous reservoir compared to that in the systemic circulation to achieve efficient targeting and escape systemic toxicity [14]. We examined the skin delivery and anti-inflammatory activity of aglycone and glycoside flavonoids to select the potential candidates for topical application with the aim of prevention or therapy. The aqueous vehicle was used as the flavonoid donor since most attempts for modeling SPR had been based on the data of aqueous solution application [15]. Naringenin and kaempferol were efficacious for topical application with respect to targeting into the skin and possible treatment capability based on the data of *S* value and TI, respectively. The cutaneous permeation of flavonoids exhibited a similar trend either at an equivalent dose or at the saturated solubility. Both aglycones also produced a protective effect on skin-barrier function. The glycosides generally showed lower absorption than the corresponding aglycones. Previous study [16] also suggests a low skin bioavailability of flavonoid glycosides such as baicalin.

The physicochemical properties of flavonoids from the experimental data and molecular modeling prediction were beneficial to discussing the SPR. The aglycone lipophilicity was decreased following the increase of the hydroxyl group number. An exception was naringenin, which had only 3 hydroxyl groups but low lipophilicity. Different from the flavonols and flavones, naringenin is a flavanone with fewer double bonds in the C ring. The flavonols were more lipophilic and assumed a planar structure [17]. The flexibility of the chiral center in the C ring of naringenin could fold to form a three-dimensional structure. The approximation of the B ring to the A and C rings increased the possibility of the production of intramolecular hydrogen bonds, leading to the decrease of lipophilicity and enhancement of aqueous solubility. This effect did not occur with flavonols and flavones due to the rigidity of the structure.

Some physicochemical factors of penetrants can influence the skin permeation. These include lipophilicity, solubility, release rate, MW, and hydrogen bonding. The penetrants with higher lipophilicity can increase the skin-absorption capacity due to the facile partitioning and entrance into the SC lipids [18]. Both log *K*’ and log *P* are the parameters of lipophilicity. Another parameter for anticipating the skin absorption is the total polarity surface, which correlates inversely with lipophilicity and biomembrane absorption [19]. The correlation coefficient between log *P* and the total polarity surface for the flavonoids was 0.9003. The polar surface area is defined as the total sum of the surface of the polar atoms [20]. The highest total polarity surface of myricetin could explain the low skin absorption of this compound. However, the lipophilicity could not explain the high skin deposition of naringenin and kaempferol. There was no correlation between lipophilicity and skin absorption of the flavonoids. The penetrants should first partition into the SC before diffusion across the SC. The sebum spread on the SC surface contributes to a capacity-governing penetrant partitioning from the vehicle to the SC [21]. Theoretically, this partitioning is highly dependent on the lipophilicity [22]. Our results showed that the skin deposition enhancement after sebum removal was less for naringenin and kaempferol than for the other flavonoids. This suggests an ease in the partitioning of naringenin and kaempferol to the SC layer, and sebum was a barrier for the permeation of the other flavonoids. From these data, it can be concluded that the cutaneous absorption of flavonoids cannot be solely ascribed to their lipophilicity. The skin more facilely absorbed the flavonoids with moderate hydrophilicity and aqueous solubility.

Although a lipophilic nature is required for transport across the SC, a hydrophilic property is also needed for entry into the viable skin [23]. The penetrants with the log *P* > 3 show a decrease of viable-skin diffusion following the increase of lipophilicity [24]. Both the SC and the viable epidermis/dermis are the permeation barriers. The role of viable skin in flavonoid absorption could be understood by the permeation via SC-stripped skin. Myricetin and quercetin were the flavonoids with low skin absorption. The SC removal largely promoted the deposition of both flavonoids by >30 fold. This indicates a significant barrier function of the SC for the two penetrants. The skin deposition of myricetin and quercetin was 0.4 and 1.2 nM/mg after the SC’s removal, respectively. This value was still lower than the naringenin deposition after stripping. Baicalein was a lipophilic flavonoid with facile entrance into the SC because of the limited enhancement of skin deposition after stripping. However, the baicalein deposition in the stripped skin was only 0.2 nM/mg. These results demonstrate that the viable skin could be a barrier to the flavonoid permeation. The cellulose membrane is like viable skin due to its hydrophilic property. We found that the released percentage across the cellulose membrane was lower for myricetin, quercetin, and baicalein than for naringenin and kaempferol, confirming the diffusion barrier of viable skin. Naringenin and kaempferol could conquer the viable-skin barrier, resulting in the effortless absorption. The much higher aqueous solubility of naringenin compared to the other penetrants was the reason for the easy diffusion into the viable skin. Although kaempferol exerted a comparable lipophilicity to baicalein, the considerable solubility of kaempferol had led to greater absorption than with baicalein. Because of the existence of sugar moiety, the glycoside flavonoids had good solubility in the aqueous vehicle. Although the glycosides might easily permeate into viable skin according to the high percentage of release, the absorption of glycosides was less compared to that of the aglycones. The SC was the main barrier to retard glycoside delivery since SC removal could greatly enhance their deposition.

The SC layer contains lipids and corneocytes cross-linked by keratin. The SC lipids and keratin contribute to the nonpolar and polar pathways for permeation. Similar absorption should be observed for the permeation into SC-stripped skin and delipidized skin if the penetrants predominantly transport via the nonpolar route [25]. Our data supported the lipid pathway being the main diffusion mechanism for the flavonoids. Although proteins occupy 80% of the SC constituents, the polar pathway is not the major route for flavonoids because of the limited skin deposition enhancement after protein denaturation. A high affinity of the penetrants to the SC lipids can generate a reservoir in the skin for plentiful absorption [26]. To obtain detailed information on the absorption of flavonoids, we established the possible flavonoid-lipid interaction employing molecular docking. The lipid content of the SC basically comprises ceramides, fatty acids, cholesteryl sulfate, and cholesterol [27]. There are nine classes of ceramide identified in the SC [28]. We chose ceramides II, III, and VI for docking because of their abundance in human SC [29]. Palmitic acid was used as the model fatty acid. Cholesteryl sulfate presented much stronger interaction with the flavonoids than the other SC lipids. Naringenin and kaempferol showed the highest negative CDOCKER for interacting with cholesteryl sulfate. This result coincided with the highest skin deposition of both compounds. The correlation coefficient between nude mouse skin deposition and CDOCKER of aglycones was 0.9673. The negative CDOCKER of glycosides was inferior to that of aglycones with respect to interacting with cholesteryl sulfate. A contrary result was detected for the other lipids. The aglycones even showed no interaction with cholesterol. Thus, cholesteryl sulfate could offer the most suitable flavonoid-lipid interaction for predicting flavonoid absorption. The CDOCKER of other lipids such as ceramides are infeasible to be the indicator of flavonoid absorption because of the low correlation between the energy and skin deposition. For example, rutin had the highest negative CDOCKER with ceramides and fatty acid but a low skin delivery capability.

The hydrogen bond acceptor is another factor that inversely correlates with cutaneous absorption. Since the SC is the hydrogen bond acceptor [30], an increase in the number of hydrogen bonds reduces the permeation across the SC [31]. Myricetin is the compound with a higher hydrogen bond acceptor number than the other aglycones. The hydrogen bond acceptor number of glycosides was much higher than that of aglycones. Myricetin and glycosides were inferred to have less interaction with the SC according to hydrogen bonding and cholesteryl sulfate docking. Molecular size rather than lipophilicity would impact skin absorption if less interaction was detected between the penetrants and the SC components [32]. The skin diffusion is size-dependent, with the larger penetrants demonstrating lower skin delivery. Both MW and molecular volume illustrated a larger size of myricetin and glycoside structures. The consequence was the low absorption of these compounds. The molecules partially associated with the solvent cage when they traversed the skin from the aqueous vehicle. The permeation rate involved not only the molecule itself but also the entire solvated complex [33]. Myricetin and the glycosides with a higher hydrogen bond number might attract more water molecules to produce a large solvation complex, causing a detrimental effect on skin transport. Although the MW and molecular volume of naringenin were not the lowest, the folding character of this flavanone might generate a smaller size than the flavonols and flavones with a planar structure. The small molecular size after bending favored the diffusion into the skin reservoir.

The aim of topically applied flavonoids was to offer better targeting to the skin with minimal systemic absorption. The *S* value is a parameter for judging the skin-targeting efficacy. With respect to aglycones, naringenin and kaempferol showed efficient targeting to the skin tissue. The high *S* value of myricetin and quercetin was due to there being no or negligible flux. The low cutaneous deposition of myricetin and quercetin might not elicit significant bioactivity on the skin. Rutin presented a high *S* value in the case of the equivalent dose, which could favor the cutaneous efficacy. In the case of a saturated dose, baicalin had the highest *S* value among the glycosides. As with myricetin, the low deposition has limited the topical application of glycosides for therapeutic use.

Neutrophils act as the predominant phagocytic cells for the first line of defense. Although the activation of neutrophils enhances immunity to retard xenobiotic invasion, the overwhelming stimulation contributes to inflammatory disorders and adaptive immune responses [34]. Chronic inflammatory skin diseases such as psoriasis and photoaging can be characterized by neutrophil infiltration [35]. The activated neutrophils damage the skin by the production of reactive oxygen species (ROS). A respiratory burst of neutrophils is an oxygen-dependent process leading to the formation of ROS. The protective effect of flavonoids on the skin is directly linked to the antiradical potency [7]. Our results revealed a flavonoid-driven mitigation of O_2_^•–^ in fMLF-stimulated neutrophils. The O_2_^•–^ inhibition was the highest for baicalein. Although baicalein reduced O_2_^•–^ more effectively, it only exerted moderate TI due to the low efficiency of cutaneous absorption. The lowest superoxide inhibition was found for naringenin. However, this flavanone still exhibited the highest TI because of the efficacious skin targeting. The intramolecular hydrogen bond can suppress antioxidant activity [36]. This is the reason for the low O_2_^•–^ inhibition by the flavanone.

The number of hydroxyl groups in the structure of the antioxidants is a factor influencing the antiradical effect, with the presence of more hydroxyl groups resulting in stronger activity [37]. This was not the case in our study since no correlation was observed between the hydroxyl moiety number and the scavenging capacity. The O_2_^•–^ inhibition of glycosides was similar to their corresponding aglycones. The lower skin deposition of glycosides had resulted in the lower TI of O_2_^•–^ compared to the aglycones. Both neutrophil trafficking and elastase release are elevated in skin photoaging. Elastase plays a role in wrinkling after photoaging [38]. The experimental data provided evidence that the flavonoids showed less inhibition on elastase than O_2_^•–^. The glycosylation decreased the inhibitory activity on elastase. The same as O_2_^•–^, the greatest TI of elastase proved to be topically applied naringenin.

The ΔTEWL data demonstrated that the flavonoids did not damage the skin barrier as compared to the aqueous vehicle control. No SC disruption was observed for naringenin and kaempferol, though the skin deposition was very high. It is surprising that naringenin and kaempferol even alleviated ΔTEWL. Water contact with the SC in high content can disturb the barrier’s nature [39]. The aqueous vehicle used in this study could increase TEWL. The skin histology demonstrated fewer SC layers of the aqueous medium control than the sham group. Naringenin and kaempferol with high cutaneous absorption might protect the skin to reduce the impairment generated by the water. Further work is needed to elucidate the protection mechanism. Naringenin, extensively contained in lemons, oranges, and grapes, is reported to be a potent active of attenuating skin inflammation and oncogenesis [4,40]. Kaempferol has been demonstrated to restrain photoaging and UV-induced carcinogenesis [41]. The synthetic drugs such as indomethacin and celecoxib provide protection against photoaging and skin cancers but have severe adverse effects. Topically applied naringenin and kaempferol can be promising candidates as alternatives for skin-inflammation treatment.

## 5. Conclusions

This study examined the cutaneous absorption and anti-inflammatory activity of flavonoids with the goal of choosing the optimal candidates for topical delivery. The results revealed that the glycosides showed less absorption and targeting than the aglycones. The low lipophilicity and large molecular size of glycosides contributed to the unsatisfied absorption. Naringenin and kaempferol were the compounds with the greatest deposition in the skin. They also protected the skin from the barrier disruption induced by the aqueous medium. Both flavonoids are potential candidates for topical application to treat skin inflammation. Both the SC and viable skin were the permeation barriers for flavonoid transport. The SPR demonstrated the importance of the hydrophilic and lipophilic balance of the flavonoid structure in order to achieve feasible skin delivery. The hydrogen bond acceptor and docking calculated from molecular modeling described the cutaneous absorption trend. We had utilized the aqueous vehicle as the flavonoid formulation. It is expected that the different compositions of vehicle may largely affect the skin permeation of the penetrants. The flavonoids may exhibit different permeation trend in other vehicles such as oil and nanoparticles. Further study is needed to elucidate the impact of different vehicles on flavonoid absorption. Our results offer essential information for the development of a topically applied flavonoid formulation. The experimental profiles in this study also provide the direction to design or synthesize new compounds for facilitating skin delivery.

## Figures and Tables

**Table 1:** Physicochemical properties of aglycone and glycoside flavonoids.

Category	Compound	MW (Da)	log *K’*	log *P*	Solubility in 20% PEG400 (mM)
Aglycone	Myricetin	318.24	0.34	3.18 ± 0.07	0.53 ± 0.01
	Naringenin	272.25	0.51	3.21 ± 0.04	3.62 ± 0.28
	Quercetin	302.24	0.51	3.32 ± 0.09	0.38 ± 0.0025
	Kaempferol	286.24	0.65	3.71 ± 0.57	0.19 ± 0.0047
	Baicalein	270.24	0.73	3.59 ± 0.08	0.0077 ± 0.0019
Glycoside	Naringin	580.54	0.19	0.15 ± 0.04	>20
	Rutin	610.52	0.24	0.76 ± 0.22	1.84 ± 0.04
	Baicalin	446.36	0.42	0.89 ± 0.02	5.32 ± 0.15

MW, molecular weight; log *P*, partition coefficient measured by *n*-octanol/water partitioning; log *K*’, logarithm of (t_r_ − t_0_)/t_0_, t_r_ is the retention time of compound peak, t_0_ is the retention time of solvent peak. Each value represents the mean and S.D (*n* = 4).

**Table 2:** The inhibition percentage (%) of superoxide anion (O_2_^•–^) and elastase and the therapeutic index (TI) of aglycone and glycoside flavonoids at 3 µM.

Category	Compound	O_2_^•–^ Inhibition	TI_superoxide_	Elastase Inhibition	TI_elastase_
Aglycone	Myricetin	42.07 ± 6.22	0.58	16.81 ± 4.76	0.23
	Naringenin	19.72 ± 8.23	7.36	17.97 ± 6.62	6.71
	Quercetin	26.44 ± 8.07	0.89	28.67 ± 8.51	0.96
	Kaempferol	26.49 ± 8.41	2.97	18.11 ± 9.01	2.03
	Baicalein	77.48 ± 5.27	2.60	12.25 ± 2.15	0.41
Glycoside	Naringin	21.51 ± 2.53	1.59	13.89 ± 9.67	1.02
	Rutin	33.87 ± 8.67	0.39	13.16 ± 3.39	0.15
	Baicalin	75.73 ± 6.21	0.53	0	0

TI is calculated by the multiplication of inhibition percentage at 3 µM and nude mouse skin deposition at equivalent dose (6 mM). Each value represents the mean and S.D (*n* = 3).

**Table 3:** The hydrogen bond number, total polarity surface and molecular volume of aglycone and glycoside flavonoids determined by molecular modeling.

Category	Compound	Hydrogen Bond Acceptor Number	Hydrogen Bond Donor Number	Total Polarity Surface	Molecular Volume
Aglycone	Myricetin	8	6	147.68	225.35
	Naringenin	5	3	86.99	204.42
	Quercetin	7	5	127.45	217.11
	Kaempferol	6	4	107.22	203.74
	Baicalein	5	3	86.99	195.85
Glycoside	Naringin	14	8	225.05	437.66
	Rutin	16	10	265.52	444.18
	Baicalin	11	6	183.21	316.93

**Table 4:** The negative CDOCKER energy of aglycone and glycoside flavonoids to interact with the stratum corneum components determined by molecular modeling.

Compound	Ceramide II	Ceramide III	Ceramide VI	Palmitic Acid	Cholesteryl Sulfate	Cholesterol
Myricetin	−19.454	−25.852	−22.285	−19.048	−117.792	-
Naringenin	−19.143	−25.670	−24.231	−18.155	−121.188	-
Quercetin	−18.928	−21.850	−22.063	−18.032	−118.533	-
Kaempferol	−21.053	−20.831	−19.639	−17.399	−119.485	-
Baicalein	−18.841	−20.637	−20.427	−18.073	−118.632	-
Naringin	−27.144	−28.937	−37.266	−17.326	−109.196	-
Rutin	−33.007	−35.341	−38.940	−23.502	−109.365	−47.1637
Baicalin	−24.573	−30.721	−28.555	−19.751	−112.425	−53.8485

- Means no interaction.
